# Phage Therapy in Gastrointestinal Diseases

**DOI:** 10.3390/microorganisms8091420

**Published:** 2020-09-16

**Authors:** Beatriz Gutiérrez, Pilar Domingo-Calap

**Affiliations:** 1 Department of Genetics, Universitat de València, 46100 Valencia, Spain; gulobe@alumni.uv.es; 2 Institute for Integrative Systems Biology, I^2^SysBio, Universitat de València-CSIC, 46980 Valencia, Spain

**Keywords:** bacteriophage, microbiome, virome, dysbiosis, phage therapy, gastrointestinal tract

## Abstract

Gastrointestinal tract microbiota plays a key role in the regulation of the pathogenesis of several gastrointestinal diseases. In particular, the viral fraction, composed essentially of bacteriophages, influences homeostasis by exerting a selective pressure on the bacterial communities living in the tract. Gastrointestinal inflammatory diseases are mainly induced by bacteria, and have risen due to the emergence of antibiotic resistant strains. In the lack of effective treatments, phage therapy has been proposed as a clinical alternative to restore intestinal eubiosis, thanks to its immunomodulatory and bactericidal effect against bacterial pathogens, such as *Clostridioides difficile* in ulcerative colitis and invasive adherent *Escherichia coli* in Crohn’s disease. In addition, genetically modified temperate phages could be used to suppress the transcription of bacterial virulence factors. In this review, we will highlight the latest advances in research in the field, as well as the clinical trials based on phage therapy in the area of gastroenterology.

## 1. Introduction

The human body is hosting a challenging consortium of microorganisms comprising viruses, bacteria, archaea, fungi, and protozoa. These communities perform important functions that have strongly impact on our health, including enhancing our immune system, providing protection against pathogens, or even helping us to digest food [1,2]. The viral fraction forming the microbiome, called the virome, is dominated by bacteriophages [3]. Commonly called phages, they are viruses that infect bacteria, and play a crucial role in the shape of bacterial communities in most environments. Interestingly, phages are the most abundant entities in Earth, representing a major source of biodiversity [4]. In general, phages can replicate via lytic, lysogenic, chronic or pseudolysogenic cycles. Once a virion recognizes the host cell, a phage receptor-binding protein triggers the insertion of its own genome into the cell. For lytic or virulent phages, replication of the phage genome is followed by its assembly into phage particles and lysis of the host to release progeny. Lysogenic cycles occur when the phage genome is integrated into the host chromosome. Integrated phage genomes are called prophages, and the bacteria that contain them are called lysogens [4]. By incorporating its genome into the bacterium, these temperate phages can induce a change in the phenotype of the host cell, providing advantages through gene transfer that improve host virulence and resistance to immune defenses, a process called lysogenic conversion. In addition to these classical cycles, chronic cycle and pseudolysogeny have been proposed as alternative pathways. Under the chronic cycle, the phage replicates and virions are produced without killing its host. Pseudolysogenesis is a stage in which the phage genome does not multiply (as in the lytic pathway), neither replicates in a synchronized and stable manner during the cell cycle (as in the lysogenic pathway), but remains within the host as an episome, independent from the host genome [5].

After their discovery a century ago, phages have been used as therapeutic tools since the very beginning. Despite their success in the first trials conducted by Felix d’Herèlle in 1921 in patients with dysentery [6], phage therapy was highly controversial and was not widely accepted. In parallel, the discovery of antibiotics in the 1930s further diminished the enthusiasm for phage therapy in Western countries, being reduced to a few Eastern European countries. In addition, the lack of legislative regulation associated to the poor interest from the pharmaceutical industry, makes that there are not many commercial phage preparations in Europe or the USA for clinical use. However, the Eliava Institute of Bacteriophages, Microbiology and Virology (Georgia), founded in 1923, is considered a leading organization in phage research, production of phage preparations, and their practical application. Unfortunately, these treatments are not yet regulated in the Western countries, although they are currently used as compassionate treatment. Interestingly, clinical trials are rising worldwide, and in addition to the compassionate use of phages, encouraging results are fostering phage therapy as a real alternative to combat pathogenic bacteria [7]. Regulation for the use of phages as therapy involve strictly lytic phages against the causative target pathogen [8]. Thus, temperate phages are usually not recommended because of the potential acquisition of pathogenic traits or antibiotic resistance determinants through horizontal gene transfer [9]. However, advances in genetic engineering suggest the possible therapeutic use of genetically modified temperate phages to suppress the transcription of bacterial virulence factors [10].

In the last decade, thanks to advances in metagenomics and greater social awareness of the relevance of the intestinal microbiota to human well-being, research on the intestinal phagome (the phage component of the virome) has raised interest [3]. It has been observed that the coexistence of bacteria and resident phages contributes to the preservation of homeostasis in the body [11]. Intestinal phages play an important role shaping the microbiome, but also by interacting with the human immune system, creating a balance between health and disease. Under dysbiosis, alteration of the microbiota can lead to pathophysiological processes such as inflammation and immune activation. Dysbiosis in the gastrointestinal tract results mainly in Crohn’s disease (CD) and ulcerative colitis (UC), chronic recurrent inflammatory bowel disorders mediated by unregulated immune responses to the resident microbiota [12]. They are multifactorial diseases determined by genetic and environmental risk factors, including the microorganisms living in the gut [13]. In conditions of dysbiosis, microbiome is altered, resulting in a decrease of symbiotic species and/or an increase in pathogenic ones [14]. Existing approaches to modulate the intestinal microbiome include dietary changes and antibiotics [15]. However, antibiotic treatments are becoming less effective due to the emergence of multi-drug-resistant bacteria. The current situation requires the urgent implementation of new therapeutic strategies against infectious diseases, which has brought phage therapy as an interesting alternative. In gastroenterology, phages have been principally explored as promising tools in infectious diseases including cholera, and it is being tested against *Clostridioides difficile* colitis, and the eradication of adherent invasive *Escherichia coli* (AIEC) in CD [16,17]. The aim of this review is to analyze the latest scientific advances in phage therapy in the field of gastroenterology, highlighting its importance as a therapeutic tool [18].

## 2. Role of Phages in the Human Gastrointestinal Tract

The viral component of the human microbiome has been poorly studied compared to the bacterial portion [19]. The human intestinal virome corresponds to the population of viruses associated with the intestinal microbial community. According to the high predominance of bacteria in the gut, the intestinal virome is mainly colonized by phages [3]. Classically, sequencing-based analysis suggested that the intestinal virome was mainly composed by single-stranded DNA (ssDNA) phages, mainly *Microviridae,* and double-stranded DNA (dsDNA) phages of the order Caudovirales (notably the families *Myoviridae*, *Podoviridae* and *Siphoviridae*). In 2014, metagenomic studies identified a dsDNA phage genome, called *crAssphage* (crAss—cross-assembling), as a major fraction in the intestinal microbiota. Currently, *crAss-like* phages are detectable in approximately 50% of individuals in specific human populations and accounting for up to 90% of the total viral DNA load in the stool, as is considered the most abundant phage genus in the gut [20].

### 2.1. Phages and Intestinal Dysbiosis

Most phages in the human intestine are temperate, suggesting that gut microbiome is fairly stable in the gastrointestinal tract. This idea has led to the establishment of a global intestinal phagome, showing a correlation between phages and health status, and his role in maintaining the structure and function of the intestinal microbiome [21]. This stability allows the maintenance of other microorganisms of the intestinal microbiota [22]. Interestingly, the prevalence of healthy intestinal phagome was found to be significantly altered in patients with UC and CD. Bacteriophage richness, measured by the number of taxa per sample, was showed to be higher in patients undergoing these diseases, while bacterial richness decreased concomitantly [13].

**Figure 1:**
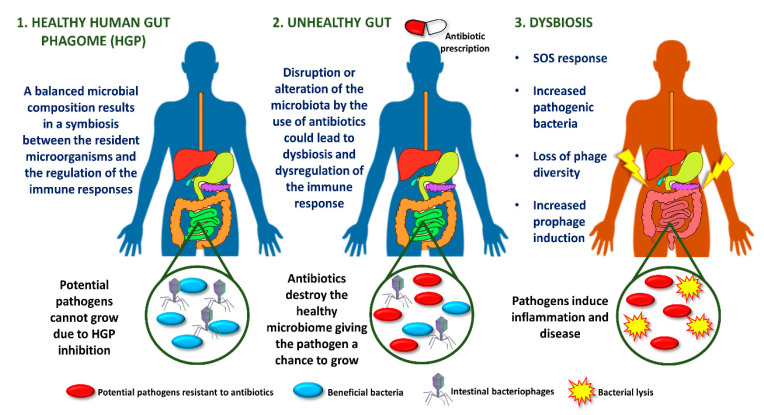
Antibiotic-associated intestinal dysbiosis.

The inflamed gut is associated with an SOS response regulated by an increase in resident intestinal pathogenic bacteria, loss of phage diversity, and induction of prophages. Several environmental conditions trigger the response to SOS bacterial stress, such as ultraviolet light, drugs or antibiotics [23]. As a clear example, most orally administered antibiotics alter the intestinal microbiota, promoting dysbiosis (Figure 1) [24].

Phages can contribute as vectors for horizontal gene transfer. The high induction of prophages during inflammation favors the horizontal gene transfer mechanism among its bacterial hosts, which increases the rate of genetic recombination and diversification. This process actively shapes the evolution of bacteria modifying virulence and antibiotic resistance factors. In addition, phage genes can indirectly increase bacterial toxin production, having implications in adhesion, colonization, and invasion of the immune response [24].

### 2.2. Phages as Immunomodulators

Another interesting characteristic of phages is their potential for regulation of the immune responses [25]. The immune system interacts with the microbiota by maintaining a non-inflammatory homeostasis, based on multiple mechanisms, such as a physical mucosal barrier and the secretion of antimicrobial compounds. Intestinal phages can actively remove invasive bacteria, but also can reduce local immune and inflammatory reactions, maintaining immune homeostasis [24].

The most immediate impact of phages on the immune system may be during sepsis, where the lytic activity of phages can reduce the bacterial load. Conversely, bacterial debris caused by phages can also lead to sepsis. The immunomodulatory properties of phages may lead to a partial buffering of the bacterial-induced inflammatory response or bacterial lysis [26]. Cellular phage-mediated lysis appears to be involved in the production of pathogen-associated molecular patterns (PAMP). If there is increased intestinal permeability, PAMPs may translocate and activate the immune responses [27]. Phages are able to stimulate bacterial phagocytosis by macrophages through opsonization, by making them more easily accessible to the immune system [26].

**Figure 2:**
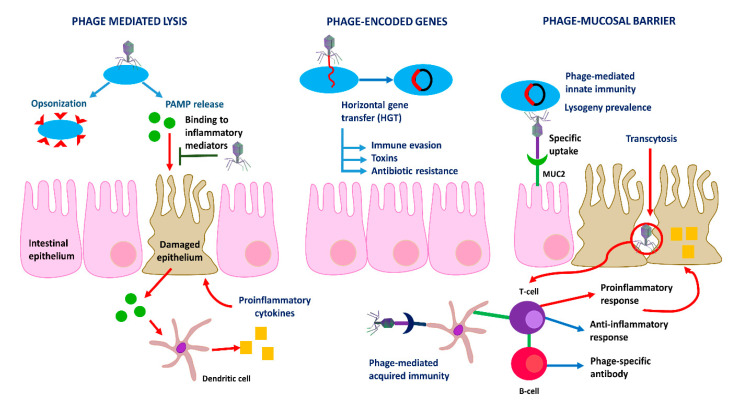
Bacteriophage-mediated immune responses.

The intestinal mucosa shapes the interactions between phages and their bacterial hosts. Phage communities establish contact with the mucosal barrier generating a phage-mediated immunity [28]. Under this model, innate immunity protects commensal microorganisms in the upper layers of mucus through lysogeny, and acquired immunity kills invasive pathogens in the deepest mucus through lysis [29]. To serve as effective antimicrobials for the host, adherent phages must reduce bacterial colonization of mucus. Several phages express proteins displaying lectin type C folds and immunoglobulin type domains, interfering with O-glycosylated MUC2 mucin in the colon [30]. For example, the outer capsid protein of the T4 phage preferably binds to the O-glucan chains in the mucins, increasing the fraction of phages in the mucosal layer, and playing a protective role against mucus-penetrating bacteria. Therefore, modification of mucosal glycosylation, could impact the abundance of certain phages, having consequences among specific bacterial groups [31]. On the other hand, a pathogen that breaks the innate immune response will be tackled by the acquired immune system. The Ig-type fold of bacteriophages is found in antibodies and T-cell receptors [26]. Moreover, phage neutralizing antibodies have been detected in the sera of different species, suggesting that phage antibodies could be common in the human population [32]. Interestingly, the limiting factor for phages in the gut is considered the production of specific immunoglobulin A (IgA). It was shown that if IgA levels were low, phages were found in feces. However, if IgA levels increased, no active phages were present in the stool (Figure 2) [26].

Therefore, the phagome shapes the bacterial populations in the gut maintaining the homeostasis. However, dysbiosis can arise, promoting the extinction or unusual growth of bacterial hosts [33].

## 3. Role of Phages in Gastrointestinal Diseases and Their Potential Clinical Outcomes

Gastrointestinal diseases are one of the most recurrent inflammatory conditions in health care, CD and UC being the two main diseases included in inflammatory bowel disease. Phages are a promising alternative to modulate the intestinal microbiota by eliminating pathogenic bacteria. Currently, lytic phage therapy is mainly being explored in gastroenterology to combat *C. difficile* UC and the eradication of AIEC in CD [17]. The most important issue for the therapeutic development of phages is to evaluate the safety and efficacy of the phages to be used. Currently, research in this field is based on in vivo animal models or using a suitable in vitro system.

### 3.1. Lytic Phages against Clostridioides difficile in Ulcerative Colitis (UC)

*C. difficile* UC is one of the most common inflammations of the large intestine that causes diarrhea. *C. difficile* is commonly found in asymptomatic people, comprising over 50% of children and 15% of healthy adults. In fact, the sole presence of *C. difficile* is not an indicator of intestinal inflammation. Disease progression actually entails the secretion of toxins such as the enzymes TcdA and TcdB, glycosyltransferases that modulate cytoplasmic Rho GTPases, impairing the integrity of the cytoskeleton. The toxin-coding region is known as the pathogenicity locus, called PaLoc [34]. PaLoc is believed to have its origin in an ancient phage, as it shares certain characteristics with phages, particularly the *tcdE* gene, which encodes a phage-like sequence involved in the secretion of the toxins. Currently, antibiotics and fecal microbiota transplantation (FMT), are used to combat *C. difficile* UC. Effectiveness of FMT has been associated to the transfer of eubiotic bacteria that restore the homeostasis [35]. A follow up was conducted on a patient with *C. difficile* infection treated with FMT for four and a half years [36]. The donor samples consisted mainly of *Bacteroidetes* and *Firmicutes*, which are characteristic of healthy intestinal microbiota. The microbiota remained different from the donor 6–7 months after FMT. Three intermediate samples contained relatively high levels of *Firmicutes*, *Proteobacteria* and *Verrucomicrobia* compared to the donor sample used for FMT. After 4.5 years, the microbiota of the patient revealed a similar composition to the donor at the phyla level, suggesting that the entire graft would require years. However, clinical symptoms were resolved immediately after FMT, suggesting that the microbiota restore metabolic functions even before the complete graft. Despite the large differences in composition over time, all the samples analyzed had comparable bacterial and viral diversity. Viral sequences were identified by metagenomics, revealing the presence of *Caudovirales* phages in all donor and patient samples tested [37]. *Caudovirales* are dominant in the human gut, followed by ssDNA phages of the family *Microviridae*. Three lytic phages were identified in all samples tested from the donor and the patient. One was the *vB_EamP-L1* phage, that infects the bacterium *Erwinia amylovora*, being probably a food contaminant. The other two phages, *Bip4v* and *B40-8*, infect bacteria of the genus *Bacteroides*, abundant in healthy humans. It was suggested that these phages were transferred from the donor to the recipient, where they could have played a key role. In addition, virome analysis did not detect pathogenic viruses in the donor or the patient [36]. Another study determined the phageome of a single donor and three patients with UC who had received FMT. FMT in all recipients resulted in 4 symptom-free weeks, and suggested that phages were the responsible of the symptom-free period. Temperate phages from the *Siphoviridaela* family were transmitted more efficiently than phages from other taxonomic groups, suggesting a competitive advantage for temperate phages. Therefore, it is important to consider phages as a key component of the microbiome in further analysis of FMT [38]. A recent study has shown that phageome alone may be sufficient to eliminate *C. difficile* infection and restore a healthy microbial structure [39]. The evaluation was performed in five patients with symptomatic CD with chronic relapse. The patients were monitored for at least 6 months and up to 33 months. Fecal filtering transplants (FFT), including viruses present in the feces but not larger components such as bacteria, were effective in treating the five patients. A total of seven phages were isolated and characterized, including morphology, host range and molecular diversity. Of these, six (*phiCDHM1* to *phiCDHM6*) were myoviruses, and one (*phiCDHS1*) was a siphovirus. When combined, they had lytic activity in 86% of the *C. difficile* clinically relevant strains tested. They demonstrated that phages could lysate 12 of 13 of the most prevalent strains in the UK. In all cases, FFT restored normal stool habits and removed *C. difficile* symptoms for at least 6 months. These results highlight that bacteriophages alone modulate the bacterial community restoring homeostasis [39].

Furthermore, the acquisition of metagenomic information about the intestinal phages is essential for the development of future specific phage therapies for pathogens in the intestine. In a recent study the bacteriome and virome of the same stool samples from 101 healthy adults have been metagenomically analyzed to understand host-bacteria-phage associations in the intestine [39]. In total, 114 prophages were successfully identified. Accordingly, metagenomic information and host bacterial associations can be used to isolate new phage-specific antibacterial compounds that could control pathogens. For example, *C. difficile* phage-specific endolysins are considered a promising infection control strategy [40].

### 3.2. Lytic Phages Against Invasive Adherent Escherichia coli (AIEC) in Crohn’s Disease (CD)

Diarrhogenic *E. coli* is a major pathogen worldwide, particularly in locations with reduced access to clean water and medical care. Among *E. coli* pathotypes, AIEC is a heterogeneous category, causing acute and persistent diarrhea [41]. Unfortunately, current treatments should be revised due to the emergence of multi-drug-resistant strains. In the last 10 years, resistant *E. coli* strains are raising fast, with extremely fast worldwide dissemination. Under these circumstances, alternative treatments such as those based on phages are needed to combat this important public health problem [15].

AIEC strains are considered pathobiotic because they promote inflammatory diseases, mainly indirectly through stimulation of the immune system. Several factors have been identified that are implicated in the presence of AIEC in the intestinal mucosa, promoting inflammation. Genes involved in AIEC virulence may enhance motility, capsule and lipopolysaccharide (LPS) expression, serum resistance, iron absorption, adhesion and invasion of epithelial cell lines and biofilm formation. Other virulence features of AIEC are linked to its replication and survival within macrophages. AIEC can exploit host apoptosis mechanisms by increasing S-nitrosylation and proteasome degradation of caspase-3 in infected macrophages. It is also worth mentioning that AIEC can induce autophage cell death in neutrophils. In the presence of antibiotics, AIEC activates the production of neutrophil ROS contributing to intestinal inflammation. Thus, AIEC can prevent an adequate antimicrobial response [41].

Several interesting investigations have been carried out to assess the use of phages to combat AIEC. LF82 strain, isolated from the ileum of a CD patient, has been commonly used for most studies involving inflammatory bowel disease-associated *E. coli*. The LF82 strain can bind to the host adhesion receptor, that is strongly expressed in the ileal tissues of CD patients. An interesting study testing phages as a potential treatment against AIEC was conducted with three phages, *LF82_P2*, *LF82_P6* and *LF82_P8*, to determine the ability of this cocktail to infect adherent LF82 bacteria in a mouse model. By 24 h after treatment, the level of the LF82 strain in the feces was two-fold decreased in the phage-treated group, remaining significantly lower than in the control group after 4 days. In addition, ileal and colon intestinal sections were tested for bacterial quantification 24 h after treatment, showing that LF82 was significantly lower in all intestinal sections of the phage-treated group than in the control. Therefore, by both direct and indirect methods, they demonstrated that a single administration of a phage cocktail reduced the level of LF82 colonization, suggesting that phage therapy could be an interesting therapeutic tool [42].

Another example using phages against diarrhogenic *E. coli* was performed using the PDX phage, belonging to the *Myoviridae* family. PDX lysed the targeted bacteria in a murine model of intestinal colonization. In vitro, it was shown that the PDX phage killed the targeted bacteria, and using 16S rDNA analysis, it was demonstrated that the α and β diversity of the microbiota was not affected. Using the mouse colonization model, a single dose of PDX decreased significantly AIEC colony counts at 2, 3, and 5 days post-infection, concluding a reduction in the presence of *E. coli* in the mouse. Therefore, PDX represents a potential solution against diarrhea, and it could be used in prevention and as a therapeutic tool [41].

### 3.3. Temperate Phages to Suppress Virulence Factors

Phage therapy encourage the use of strictly lytic phages, mainly to avoid horizontal gene transfer resistance through specialized transduction. However, the development of phage genome engineering allows to increase the safety and efficacy of temperate phages [42]. In addition, temperate phages could be modified to deliver synthetic genes in order to suppress virulence genes or to resensitize the bacterium to antibiotics. Since temperate phages are not lytic, this approximation diminishes the risk of endotoxin release [43].

For example, temperate phages have been engineered to introduce *rpsL* and *gyrA* genes, that confer sensitivity to two antibiotics, streptomycin and nalidixic acid. Modified λ phage containing the *rpsL* and *gyrA* genes allows, after its integration, to restore sensitivity to the two antibiotics. However, this kind of approximation is still limited, and proper implementation may require the use of a non-toxic form of selective pressure [43].

An interesting example of the use of genetically modified temperate phages was done to suppress the Shiga toxin (Stx) from an established *E. coli* population that colonizes the intestine of mammals, a pathogenic infection difficult to treat. Although antivirulence drugs targeting the toxin have been investigated, clinical trials have failed [44]. The *stx* gene encoded by the temperate 933W phage is expressed after the induction of the lytic cycle. In the prophagic state, 933W remains latent through the expression of its repressor protein, cI. After induction, the bacterial SOS response induces RecA-mediated degradation of cI, leading to the expression of lytic genes that induce both, phage progeny and the expression of *stx*. Genetically modified temperate phage λ (933W·cI ^ind-^) has been obtained with the ability to neutralize Stx production, and capable of overcoming phage resistance mechanisms. The engineered phage was showed to infect, lysogenize, and inhibit Stx production from *E. coli* in vitro and in vivo in a murine model [11].

## 4. Phage Therapy: Clinical Trials in Gastrointestinal Diseases

Randomized controlled clinical trials in humans are required to evaluate the potential of phage therapy in gastrointestinal health. So far, a few human clinical trials with bacteriophages have been conducted to treat gastrointestinal diseases.

A prospective single-center randomized clinical trial was conducted to determine the safety and efficacy of a T4-like coliphage cocktail [45]. The cocktail or placebo was administered orally for 4 days to children with acute onset dehydrating diarrhea of less than 48 h duration. The safety of the cocktail was evaluated clinically and by functional testing. The cocktail comprised 11 lytic T4-like phages (*AB2*, *4*, *6*, *11*, *46*, *50*, *55*, *JS34*, *37*, *98*, *D1.4*), lacking bacterial virulence genes. The placebo was a standard treatment, an oral rehydration solution supplemented with zinc [45]. The results showed that the T4-like phage cocktail did not show a clinical improvement over the placebo in terms of fecal output, frequency or rehydration. Digging deeper into the data, only 60% of the 120 patients enrolled had a stool-borne *E. coli* pathogen, targeted by the phage cocktail, and in those patients, less than 5% of total fecal bacteria were *E. coli*. To overcome the limitations of future pharmacotherapy trials, the authors strongly recommend controlling whether the target pathogen infects different strains to support in vivo phage replication. Although this trial was not able to conclude the treatment activity, it was interesting to validate safety issues of phage therapy. Notably, no substantial in vivo replication of the orally applied phage was observed. Overall, the oral administration of the cocktail showed safe intestinal transit in children. However, did not treated the symptoms, probably due to the low *E. coli* abundance [45].

In addition, a randomized, double-blind, placebo-controlled crossover intervention was performed to assess the safety and tolerability of phages in healthy adults suffering mild to moderate gastrointestinal distress [46]. Using a commercial cocktail containing four phages (*LH01-Myoviridae*, *LL5-Siphoviridae*, *T4D-Myoviridae* and *LL12-Myoviridae*) targeting *E. coli*, the study aimed to determine their effects on the gut microbiota, including markers of intestinal and systemic inflammation. Chronic diarrhea is associated with low blood tCO_2_ levels, and the results showed a significant increase in tCO_2_ after treatment. In addition, aspartate aminotransferase and alanine aminotransferase levels in samples collected after treatment were lower compared to placebo. It is known that these enzymes levels increase after exposure to bacterial lipopolysaccharides, associated with systemic inflammation and tissue damage. The phages in the cocktail target pro-inflammatory *E. coli*, and it is possible that phages reduce circulating endotoxin by modulating intestinal microbiota and intestinal barrier function. In addition, a reduction in the circulation of cytokines was detected, probably associated to a reduction in the circulating bacteria due to the phage treatment, since LPS is associated with systemic inflammation and cytokine release [47]. In addition, reductions in *E. coli* fecal loads were observed in phage-treated patients but with similar diversity parameters, suggesting that the phages maintain the microbiota. In conclusion, the clinical study concluded that oral administration of phages was safe and tolerable in humans affected by moderate gastrointestinal symptoms [48].

Finally, an ongoing clinical trial targeting AIEC in CD patients is now in phase 2. This double-blind, placebo-controlled clinical trial is assessing safety and effectiveness of a phage cocktail (EcoActive) in a cohort of 30 CD patients. This trial is registered in ClinicalTrials.gov number (NCT03808103).

To conclude, although not related to gastrointestinal infectious diseases, it is worth mentioning the evaluation of phage therapy as a safe treatment in a recent clinical trial against severe infections, such as endocarditis and septic shock, caused by *Staphylococcus aureus*. A phage cocktail of three lytic phages (*Myoviridae*) was used to treat patients with *S. aureus* bacteremia. An intravenous administration was performed for two weeks, twice a day, and the patients’ safety and tolerance to the phages were evaluated. The results indicated that the phage preparation was safe in severe *S. aureus* infections, eliminating bacteremia and reducing inflammation. Interestingly, no bacterial resistance to phages was observed [49].

## 5. Conclusions

Gastrointestinal phagome is an emerging field of research that will have important implications in the understanding of gastrointestinal diseases. Determining ecological key parameters of phages in the gut will provide interesting insights about their role as modulators of the homeostasis. Therefore, the identification of a healthy phagome will afford an exciting approach to developing new treatments. In this view, preliminary evidence showing the contribution of phages to microbial resilience and recovery of patients through fecal filtering transplants is encouraging. Moreover, the development of animal models that mimic the intestinal community will be useful to test the impact of phages in the bacterial communities. In addition, synthetic biology allows the use of temperate phages for therapeutic purposes, opening a new area of phage-based research.

In conclusion, phages contribute to the structure and function of our human intestinal microbiota, influencing states of gastrointestinal health and disease. Under this scenario, phages should be considered as a promising therapeutic tool against pathogenic gastrointestinal bacteria. However, there is still a lack of standardization and legal frameworks that must be addressed. Encouraging future research in the field will therefore contribute to establishing regulatory and safety protocols, resulting in the use of phages as clinical tools. Therefore, additional and more in-depth studies in the field will further enhance our current knowledge of the role of phages in the microbiome and will provide a solid basis for future therapeutic implications.
